# Blood Condition a Controlling Factor in Extractions*This is an extract from a paper by Dr. Hartzell, entitled, “A Discussion of the Factors to be Considered in Determining Whether to Extract or Conserve Diseased Teeth,” published in full in the December *Journal N. D. A.*

**Published:** 1920-01

**Authors:** Thomas B. Hartzell


### BLOOD CONDITION A CONTROLLING FACTOR IN EXTRACTIONS *

BY THOMAS B. HARTZELL, D.D.S.

By a study of the flora of the mouth from the period of eruption of teeth until adult life the streptococcus makes practically fifty per cent. of the mouth growth. We now know that the presence of the streptococcus is almost universal. It occurs in houses, in dust, on the skin, in milk, and in many foods. There have been at least three streptococcal epidemics produced through the medium of milk, which have been reported in the medical journals. Probably, the most striking one was that in Boston, which was described by Theobald and Smith in the *Journal of Medical Research*, and has been alluded to by many other writers.

The individuals who advocate complete destruction of dental organs in order to rid the body of streptococcal

* This is an extract from a paper by Dr. Hartzell, entitled, "A Discussion of the Factors to be Considered in Determining Whether to Extract or Conserve Diseased Teeth," published in full in the December *Journal N. D. A.*

growth, therefore evidence profound ignorance. Wholesale extraction of teeth will not rid the body of streptococcal growth. One might as well advise removal of the transverse colon to rid the body of the colon bacillus. A much more sane method of cutting down the inroads of mouth infection is that of vigorous systemic mouth sanitation, together with the adoption of a diet planned to prevent intestinal putrefaction. This is just as capable of reducing the enormous mouth growth to a safe minimum as are the present methods for controlling bacterial growth in milk, now demanded by our cities. The difference by weight and count in the number of bacteria per milligram of tooth scrapings in dirty mouths to that of reasonably clean mouths has been shown by Kligler and Gies to range from eight hundred million in the dirty mouth to five to eight million in the clean mouth. Translated into terms of oral prophylaxis, we may find that ten minutes a day spent in proper mouth sanitation will produce this safe minimum. The writer's first thought then in regard to the extraction question is that correct mouth sanitation and proper diet will make ruthless extraction of teeth unnecessary. A proper education of the dental profession in a broad way will soon enable them to place in a true perspective the factors which actually justify conservation, or, on the other hand, extraction.

Please recall that the mouth streptococcus makes its appearance at birth, and that it forms (roughly speaking) half the mouth growth; also, that our bodies are of greater or less degree, sensitized to its presence and that its most destructive attribute is the production of secondary anemia. Its toxins lessen vitality and lay a foundation of physical weakness in an individual for its ravages in the tissues when it becomes too numerous for the leucocytes in the blood stream to destroy the individual members of the family, which gain access to the stream. Therefore, the first broad view that should come into the mind of an individual as to the conservation or destruction of infected teeth should rest on the wealth or poverty of the blood stream. To illustrate, an individual with a full complement of red cells, five million to the cubic centimeter of blood with a hemoglobin of not less than eighty-five, and a leucocyte count of seven to eight thousand to the cubic centimeter together with a normal digestion, a normal urine, and a reasonable amount of energy, is safe in conserving any of his dental organs which might present for treatment. This conservation should be, first, the reduction of mouth growth by prophylactic means to as nearly as possible the minimum, and, where teeth must be devitalized, such devitalization should be done under exactly the same kind of surgical asepsis, as is demanded of surgery of the brain or abdominal cavity.

Now, as to the other side of the question, as to when we are justified in breaking down dental mechanisms by extraction. This must rest again upon an examination of the patient's vital resistance, expressed by the blood stream, together with any evidence which tends to prove that the individual has been oversensitized or brought into a condition of anyphylaxis to streptococcal infection by a long exposure to such infection. A clinical picture, which would demand extraction, might be as follows: Early history of tooth and tonsil infection, swollen glands, draining the mouth and throat area, poverty of red cells, reduced hemoglobin, and a markedly increased or decreased leucocyte count. It is a well-recognized fact that a leucocyte count above ten thousand is strongly indicative of deep-seated infection, while a low leucocyte count, say five thousand or less, points to the fact that the leucocytes are waging a losing battle, particularly if such reduction in number is accompanied by reduced hemoglobin. If a clinical picture presents with the evidence of secondary infection, myocarditis, endocarditis, or nephritis accompanied by joint or kidney disease (evidenced by casts, albumen, or sugar) extraction is justifiable. Any of the factors above recited, accompanied by the poverty of red cells and poverty of hemoglobin, even if the leucocyte count be normal, fully justify the extraction of diseased teeth. One might go on and enumerate combinations of clinical pictures which would bring in many other physical conditions dependent upon streptococcicosis. For instance, in chronic streptococcal infection, one infrequently finds cholecystitis, and on palpitation, an enlarged spleen. While the dentist, who has not had the advantage of a medical training, may not be capable of gathering all the evidence tending to establish a condition demanding the positive eradication of all primary sources of infection which can be reached, he can always or almost always bring into his counsel, an internist or the family physician to aid him in gathering the necessary data on which to base a sane conclusion. The time is fast approaching, if not already here, when the men in the practice of medicine and dentistry are willing to listen to the arguments, pro and con, which should determine a wise course of treatment. Individuals, who present a clinical picture which tends to establish the fact that the body has waged an unsuccessful war against streptococcal infection for a series of years, perhaps from youth up, and who are now suffering from any one of the evident phases of streptococcal infection, had better exist without teeth than go on with a primary intake from even one abscessed tooth. It is my observation that many times a sensitization of years standing with a single, small continuous source of infection is sufficient to maintain such a marked secondary condition as to utterly unfit the individual for the duties of life.

We need to emphasize the fact that modern means of laboratory diagnosis and the taking of careful case histories with the modern X-ray pictures, should be the foundation stones upon which conservative or destructive dentistry should be determined.

If the dental and medical professions will get together on all cases in which extraction is advocated, and will gather and weigh the evidence each case presents, it seems altogether likely to me that we can arrive at such a sane and reasonable judgment of the conditions that justice will be done to all.

